# Neural Correlates of Cognitive-Attentional Syndrome: An fMRI Study on Repetitive Negative Thinking Induction and Resting State Functional Connectivity

**DOI:** 10.3389/fpsyg.2019.00648

**Published:** 2019-03-26

**Authors:** Joachim Kowalski, Marek Wypych, Artur Marchewka, Małgorzata Dragan

**Affiliations:** ^1^Faculty of Psychology, University of Warsaw, Warsaw, Poland; ^2^Laboratory of Brain Imaging, Nencki Institute of Experimental Biology, Polish Academy of Sciences, Warsaw, Poland

**Keywords:** repetitive negative thinking, cognitive-attentional syndrome, rumination, resting state, fMRI, neural correlates

## Abstract

**Aim:**

Cognitive-attentional syndrome (CAS) is the main factor underlying depressive and anxiety disorders in the metacognitive approach to psychopathology and psychotherapy. This study explore neural correlates of this syndrome during induced negative thinking, abstract thinking, and resting states.

**Methods:**

*n* = 25 people with high levels of CAS and *n* = 33 people with low levels of CAS were chosen from a population-based sample (*N* = 1225). These groups filled-in a series of measures of CAS, negative affect, and psychopathology; they also underwent a modified rumination induction procedure and a resting state fMRI session. Resonance imaging data were analyzed using static general linear model and functional connectivity approaches.

**Results:**

The two groups differed with large effect sizes on all used measures of CAS, negative affect, and psychopathology. We did not find any group differences in general linear model analyses. Functional connectivity analyses showed that high levels of CAS were related to disrupted patterns of connectivity within and between various brain networks: the default mode network, the salience network, and the central executive network.

**Conclusion:**

We showed that low- and high-CAS groups differed in functional connectivity during induced negative and abstract thinking and also in resting state fMRI. Overall, our results suggest that people with high levels of CAS tend to have disrupted neural processing related to self-referential processing, task-oriented processing, and emotional processing.

## Introduction

Cognitive-attentional syndrome (CAS) is a key construct in Wells’ metacognitive theory of emotional disorders (Wells and Matthews, 1994; Wells, 2009). In the Self-Regulatory Executive Function (S-REF) model, CAS is a set of psychological processes that includes repetitive negative thinking (worry and rumination), threat monitoring, and associated unhelpful behavioral and cognitive strategies; it is derived from metacognitive beliefs, either positive (e.g., “If I ruminate I will understand my situation”) or negative (e.g., “I cannot control my ruminative thoughts”). While moments of negative self-appraisal are relatively brief in most people, the prolonged occurrence of negative emotions and negative self-appraisal in some people is due to recurring activation of CAS. This specific style of responding to negative thoughts is considered a transdiagnostic factor which underlies emotional disorders. Many studies have confirmed the relationship of CAS with emotional distress as well as symptoms of mood and anxiety disorders (Fergus et al., 2012, 2013). According to the metacognitive model, CAS is a prominent factor in the development of mood disorders, e.g., major depressive disorder (MDD; Papageorgiou and Wells, 2001, 2003, 2009; Wells, 2009), anxiety disorders, e.g., generalized anxiety disorder (GAD; Wells, 1999, 2005, 2007, 2009), post-traumatic stress disorder (PTSD; Wells and Sembi, 2004; Wells, 2009; Bennett and Wells, 2010), and obsessive-compulsive disorder (OCD; Fisher and Wells, 2005; Myers et al., 2009a,b; Wells, 2009; Solem et al., 2010).

A fundamental element of CAS is a pattern of negative, pervasive, and recurring thoughts. Rumination is associated with decreased attentional resources (Donaldson et al., 2007; Koster et al., 2011), the occurrence of negative emotions, and difficulties with problem solving (Nolen-Hoeksema et al., 2008). A ruminative thinking style is most often associated with mood disorders, as it is a risk factor for the development of depression (Nolen-Hoeksema et al., 2008) and is generally associated with dysphoric and depressive mood (Mor and Winquist, 2002). However, rumination is not only present in mood disorders – it also plays a prominent role in the symptomatology of other emotional and psychiatric disorders, such as anxiety or eating disorders (Olatunji et al., 2013). Pathological worry, another form of extended thinking, is considered a key feature of GAD; however, many researchers have shown that it also occurs in other types of emotional disorders (e.g., Starcevic et al., 2007; Spinhoven et al., 2015).

To date, there have been no studies on brain functioning in people with high levels of CAS – i.e., elevated levels of CAS-related symptomatology: repetitive negative thinking, attention to threats, unhelpful coping behaviors, and maladaptive metacognitive beliefs. There are, however, some studies using functional magnetic resonance imaging (fMRI) methods in which induction of core aspects of CAS – rumination (state rumination rather than trait rumination; Cooney et al., 2010; Berman et al., 2014; Burkhouse et al., 2017) or worry (Paulesu et al., 2010) – has been employed. The first two of the aforementioned studies on rumination induction compared depressed participants to healthy controls, while the third compared adolescents with remitted MDD to healthy controls. The Rumination Induction task used in an fMRI setting by Cooney et al. (2010) consisted of alternating blocks of ruminative, concrete, and abstract sentences which participants were asked to think about (e.g., “think about the expectations people have for you”). In this procedure, ruminative sentences, in comparison to concrete/abstract sentences, were associated with altered activity in brain regions involved in emotion processing and regulation in depressed patients: the dorsolateral prefrontal cortices, cingulate cortices, amygdalae, and parahippocampi (Cooney et al., 2010). Another study compared resting state functional connectivity with functional connectivity during negative mood induction using personalized cues created by ruminating on negative autobiographical events (e.g., “Please recall a specific time when you were very embarrassed”; Berman et al., 2014). This study showed that depressed patients had stronger connections within brain regions belonging to the default mode network (DMN), like the cingulate cortex. It was suggested that these results may be understood as difficulty in down-regulating self-oriented emotional and cognitive processing after rumination induction (Berman et al., 2014). A fourth study (Burkhouse et al., 2017) found that rumination induction with prior negative mood induction (e.g., “Remember when you failed badly at something”) elicits stronger neural activations in regions involved in the DMN and emotion processing in remitted MDD adolescents. A study by Paulesu et al. (2010) explored differences in worrying between patients with GAD and healthy controls. Sentences which induce worrying (e.g., “Mull over what worries you about your future”) were related to activation in the anterior cingulate and dorsal medial prefrontal cortex in the GAD group.

Several recent meta-analyses on neuronal functioning in people with depression (Hamilton et al., 2012; Palmer et al., 2015), specific phobias (Ipser et al., 2013), and PTSD (Simmons and Matthews, 2012) show, in general, that emotional disorders are most prominently connected to the dysregulation of subcortical brain areas involved in emotion processing, i.e., the amygdalae and hippocampi, as well as the striatum. This dysregulation is interpreted as the overdeveloped salience of threatening or saddening stimuli. Also, several cortical regions are involved in this type of processing, like the insulae and dorsolateral prefrontal cortices. Studies on repetitive negative thinking induction and large meta-analyses on emotional disorders have found that people experiencing mood and anxiety disorders exhibit dysregulation of the default mode, salience, and executive networks. Overall, people with emotional disorders demonstrate a pattern of disrupted neural processing in the areas of self-referential, task-oriented, and emotional processing (Hamilton et al., 2012; Simmons and Matthews, 2012; Ipser et al., 2013; Palmer et al., 2015).

In the current study, we aimed to explore differences in neural functioning between people with high and low levels of CAS symptoms. Given that there are no previous studies on the neural correlates of CAS, we decided to base our hypotheses on available work on repetitive negative thinking induction and meta-analytical results regarding emotional disorders which, according to metacognitive theory, are undergirded by CAS. We hypothesized that people with high levels of CAS symptoms will show similar patterns of cortical activations to those found in studies on neural correlates of depressive and anxiety disorders, as described above. To test these hypotheses, we employed a modified Rumination Induction procedure and resting state functional magnetic resonance imaging (rsfMRI). We expected that differences in neural activation in people with high levels of CAS symptoms (HCAS) would be comparable to the patterns of activation reported by Cooney et al. (2010) in depressed patients, with greater neural activity in the amygdalae, hippocampi, and cingulate and dorsolateral cortices in the rumination condition as compared to the abstract condition. We also hypothesized that the cortical regions associated with rumination and which show aberrant activity in emotional disorders will show different patterns of functional connectivity in the HCAS group in comparison to the group with low levels of CAS symptoms (LCAS). We expected to find disrupted patterns of connectivity within and between several neural networks: the DMN, the salience network, and the central executive network (CEN).

## Materials and Methods

### Procedure and Sample Selection

**FIGURE 1:**
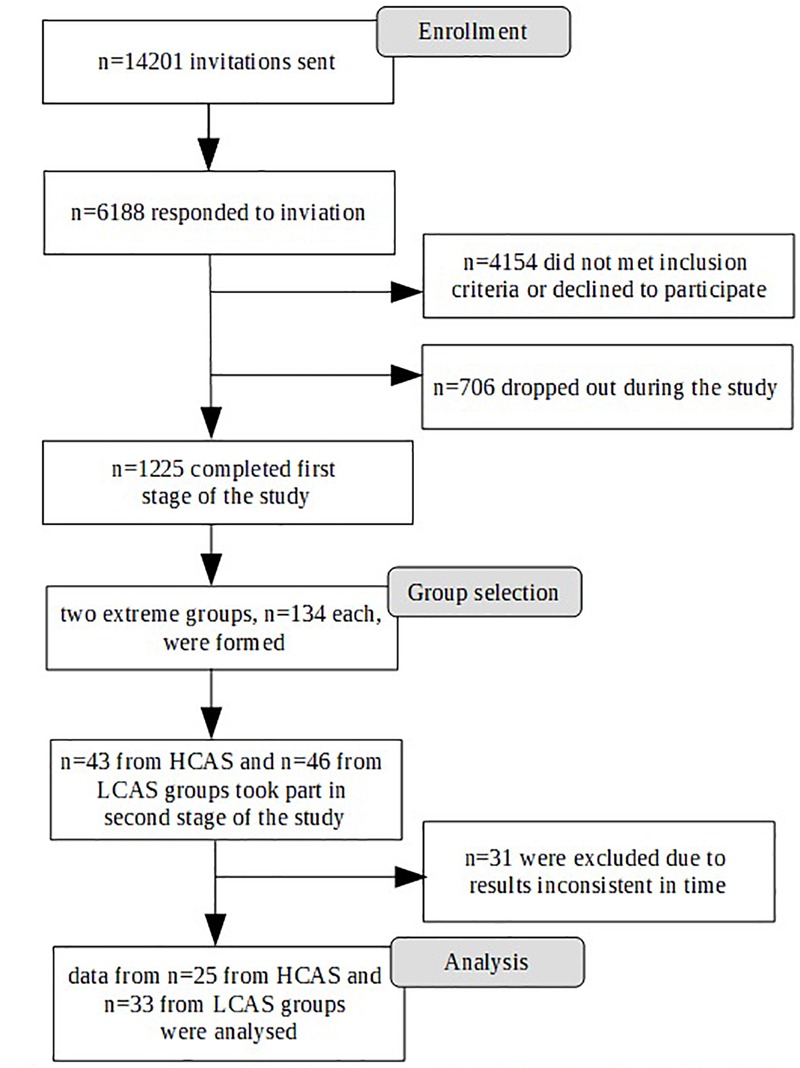
Consort flow-chart of enrollment and samples selection for the study.

Participation in the study was voluntary and participants gave their informed consent. The study was approved by the Research Ethics Committee at the Faculty of Psychology, University of Warsaw. The study was conducted in two stages. The first stage took place through an Internet survey panel and was conducted by an external company. A large sample was gathered for the purpose of an fMRI study, so there were standard strict exclusion criteria related to the fMRI procedure (left-handedness, metal objects within the body, irremovable piercings, etc.) as well as any history of neurological or serious mental disorders or substance abuse disorders. Participants were also required to live in the Warsaw area to ensure their ability to participate in the second stage of the study. A total of 1,225 participants were eligible and completed the first stage of the study. Participants were selected based on quotas mirroring the population of Warsaw (Central Statistical Office, 2017) in terms of sex, age, and education. Figure 1 depicts the selection procedure from the first to the final stage of the study.

From the first stage participants, two extreme groups were selected. As the results of previous studies (Kowalski and Dragan, 2019) have suggested that combining different measures of aspects of CAS is best for predicting levels of psychopathology, several measures were used in forming the two groups. The cut-off criterion was a score above the 66th percentile or below the 33rd percentile of the sum of results on the following measures: the CAS-1 questionnaire, the Brooding subscale of the RRS (as this aspect of rumination is most robustly associated with depressive and anxiety disorders, cf. Olatunji et al., 2013), and the Need to Control Thoughts as well as the Uncontrollability and Danger subscales from the MCQ-30, as these aspects of metacognitive beliefs are most prominently connected to levels of anxiety and depression (cf. Wells and Cartwright-Hatton, 2004; Spada et al., 2008; Dragan and Dragan, 2011; Sarisoy et al., 2014). Finally two extreme groups, each consisting of 134 subjects, were formed.

The second stage of the study took part in the Laboratory of Brain Imaging, Neurobiology Center, Nencki Institute of Experimental Biology, Polish Academy of Sciences. Participants were invited to the laboratory in a random order by a person from an external company. Researchers were blinded to the participants’ group affiliation. A total of 89 participants took part in the study – 43 in the HCAS group and 46 in the LCAS group. Participants who underwent the whole fMRI procedure were given a sum of money equivalent to about 50 EUR.

**FIGURE 2:**
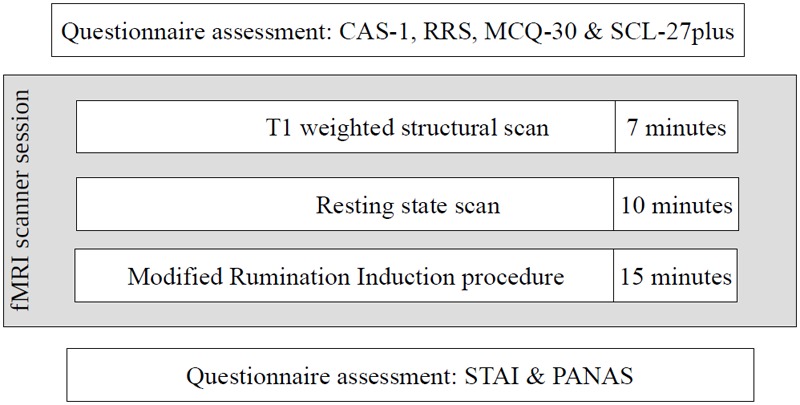
Schematic diagram of the study procedure.

The second stage of the study occurred 4–22 weeks after the first stage, depending on the timing of the participants’ second stage appointment. Despite the acceptable time-stability of the questionnaire results between the first and second stages of the study (correlations of results at these two time points: CAS-1: *r* = 0.83, *p* < 0.001, RRS – Brooding: *r* = 0.82, *p* < 0.001, MCQ – Need to Control Thoughts: *r* = 0.76, *p* < 0.001, MCQ – Uncontrollability and Danger: *r* = 0.82, *p* < 0.001) some shift in individual results was observed. To ensure that both groups had extreme characteristics, participants had to have results above or below median on all four measures used in the study. As a result, 31 participants were excluded: 30 had mixed results and 1 “changed groups” as this participant had HCAS results on the internet measures but LCAS results on the day of the fMRI scan. Ultimately, data from 58 participants (HCAS = 25, LCAS = 33) were analyzed and are presented in this paper. Group demographic characteristics are presented in Table 1. These groups were also clinically diagnosed with a SCID-I interview but full results are presented elsewhere (Kowalski and Dragan, 2019; Dragan and Kowalski, unpublished). A total of 45% of participants from the HCAS group and none from LCAS group met the diagnostic criteria for a current diagnosis of a psychological disorder. In the HCAS group, 12 participants were diagnosed according to DSM-IV-TR criteria with: MDD (1), dysthymic disorder (1), GAD (2), GAD comorbid with social phobia (1), GAD comorbid with social phobia and dysthymic disorder (1), PTSD comorbid with MDD (1), PTSD comorbid with social phobia (1), PTSD comorbid with binge eating (1), cyclothymic disorder comorbid with bulimia nervosa (1), depressive disorder NOS (1), and anxiety disorder NOS (1). All participants were treatment-naive and diagnosis-naive at the beginning of the study. The second stage of the procedure consisted of filling-in questionnaires (CAS-1, RRS, MCQ-30, SCL-27) followed by the MRI procedure, including: a T1-weighted structural scan, rsfMRI, and a Rumination Induction procedure. This MRI procedure lasted approximately 40 min in total and constituted a part of a larger MRI study. After the MRI procedure, participants filled-in PANAS and STAI questionnaires. A schematic representation of the procedure is displayed in Figure 2.

**Table 1:** Group characteristics – demographic and clinical variables.

		HCAS (*n* = 25)	LCAS (*n* = 33)	*t*-Test	*p*-Value	Cohen’s *d* (90% CI)
Sex		72% females	42% females	5.03ˆ*	0.025	0.30^∗∗^
Age		30.40 (7.26)	33.48 (5.85)	-1.74	0.089	
CAS-1		75.90 (9.98)	23.88 (11.21)	18.33	<0.001	4.90 (4.04–5.77)
RRS-brooding		15.72 (2.57)	7.09 (1.81)	14.30	<0.001	3.88 (3.15–4.62)
MCQ-30	Need to control thoughts	16.64 (2.64)	7.67 (1.51)	15.19	<0.001	4.17 (3.40–4.94)
	Uncontrollability and danger	19.28 (2.99)	8.70 (2.79)	13.87	<0.001	3.66 (2.95–4.37)
SCL-27 plus	Depression	9.63 (4.32)	0.62 (1.04)	9.99	<0.001	2.87 (2.25–3.48)
	Vegetative symptoms	8.28 (3.35)	3.88 (3.05)	5.22	<0.001	1.37 (0.89–1.85)
	Agoraphobic symptoms	4.64 (2.91)	0.52 (1.06)	6.75	<0.001	1.88 (1.36–2.4)
	Sociophobic symptoms	11.04 (2.47)	2.67 (2.17)	13.68	<0.001	3.6 (2.90–4.30)
	Pain	9.72 (3.02)	5.90 (2.45)	5.30	<0.001	1.39 (0.91–1.87)
	Total score	43.46 (9.69)	13.72 (7.06)	13.30	<0.001	3.51 (2.82–4.20)

*^∗^Chi-squared test; ^∗∗^Cramer’s Phi; CAS-1, Cognitive-Attentional Syndrome Questionnaire; RRS-brooding, Ruminative Response Scale - brooding subscale; MCQ-30, Metacognitions Questionnaire - Short Version; SCL-27 plus, Symptoms Checklist 27-plus.*

### Measures and Materials

#### The Cognitive-Attentional Syndrome Questionnaire (CAS-1)

The CAS-1 questionnaire (Wells, 2009) consists of 16 items measuring aspects of CAS: worry/rumination, attention to threat, maladaptive behaviors, and metacognitive beliefs. The results of the questionnaire were calculated as in the paper by Fergus et al. (2012) – the last eight items were recalculated to range between 0 and 8 before summing them up. The total results range from 0 to 128, where a higher result indicates a greater level of CAS. The psychometric qualities of the Polish version of CAS-1 are presented elsewhere (Kowalski and Dragan, 2019). In the current study, CAS-1 had excellent internal consistency of Cronbach’s α = 0.91.

#### Ruminative Response Scale (RRS)

The 22-item Ruminative Response Scale focuses on one’s responses to depressive mood: concentration on the self, symptoms, and the causes and consequences of depressive mood. A newer approach (Treynor et al., 2003) distinguishes two subscales: “Reflection” and “Brooding.” Only the results of the latter are presented in this study. This subscale consists of five items with results ranging from 5 to 20, where a higher result indicates a greater tendency to respond to depressed mood with brooding. The Polish version of the RRS has generally good psychometric qualities (Kornacka et al., 2016). In the current study, the Brooding subscale had internal consistency of Cronbach’s α = 0.88.

#### Metacognitions Questionnaire – Short Version (MCQ-30)

The short version of the Metacognitions Questionnaire, developed by Wells and Cartwright-Hatton (2004), consists of five subscales and 30 items. It concerns metacognitive beliefs: monitoring techniques, judgments, and beliefs about one’s thoughts and cognitive abilities central to the metacognitive model of psychopathology. Two subscales are of interest in present study: the “Uncontrollability and Danger” scale explores the negative aspects of worry, e.g., “My worrying is dangerous for me” and the “Need to Control Thoughts” scale deals with beliefs about the negative consequences of not controlling one’s thoughts, e.g., “Not being able to control my thoughts is a sign of weakness.” The Polish version of this questionnaire exhibits good psychometric qualities and is considered equivalent to the English version (Dragan and Dragan, 2011). In this study, these two MCQ-30 subscales had good internal consistencies of α = 0.89 and α = 0.84, respectively.

#### Symptom Checklist 27 Plus (SCL-27-Plus)

This is a checklist-type questionnaire that measures depressive, vegetative, agoraphobic, sociophobic, and pain symptoms (Hardt, 2008), and it allows the calculation of a global severity index (GSI). The results on each scale can range from 0 to 20, where higher scores indicate higher levels of a given symptom. In this study, the Polish adaptation of the questionnaire was used (Kuncewicz et al., 2014) and it had an excellent internal consistency of Cronbach’s α = 0.93.

#### Positive and Negative Affect Schedule (PANAS)

This is a comprehensive measure of emotions with two distinct subscales of positive and negative affect (Watson et al., 1988). In this study, a Polish adaptation of the 30-item PANAS-state questionnaire, which has good psychometric qualities, was used (Brzozowski et al., 2010). In the current study, the internal consistencies of its subscales were α = 0.82 and α = 0.80, respectively.

#### State-Trait Anxiety Inventory (STAI)

A widely used measurement of anxiety and its cognitive and vegetative components (Spielberger et al., 1970). In this study, a Polish adaptation of the STAI-state questionnaire, which has good psychometric qualities, was used (Wrześniewski et al., 2002). In the current study, the internal consistency was Cronbach’s α = 0.93.

#### Resting State fMRI

The resting state procedure consisted of a fixation cross being shown for 10 min on the MRI display (cf. Birn et al., 2013; Patriat et al., 2013). Subjects were instructed to fix their gaze on the cross and to not move.

#### Modified Rumination Induction (RumInd-M) fMRI Task

During rumination induction, participants are asked to think about sentences that are designed to induce the process of rumination (Nolen-Hoeksema and Morrow, 1993). The sentences deal with themes of the reader’s own emotions, appraisals, and experiences. In this task, we used the mix of stimuli used by Cooney et al. (2010; rumination induction) and by Paulesu et al. (2010; worry induction) to obtain a robust repetitive negative thinking effect in participants. We used the modified procedure from Cooney et al. (2010) with ruminative/worrying sentences (e.g., “Think about the opportunities you didn’t take in your life,” “Think about what worries you have about your health”; RUM), and abstract sentences (e.g., “Think about how a plant grows”; ABS) as a control condition (see Appendix 1 for all stimuli used). Participants were asked to think about sentences presented on screen and to try to clear their minds when a cross appeared on screen. Each sentence was presented on screen for 30 s and sentences were separated by 10 s of a fixation cross. Four blocks of five sentences were presented in a non-consecutive order (RUM-ABS-RUM-ABS). After each block, participants assessed their sadness, anxiety, and engagement in thinking on a 1–5 Likert scale. Results from this task are the totals of the assessments from both blocks of the same type. The task lasted about 15 min. Two parallel versions of rumination induction were used. Versions did not differ on any of the results (all values of *p* > 0.05) and administration of the versions did not differ between HCAS and LCAS groups, χ^2^ = 0.43, *p* = 0.51.

### Behavior Analysis

Internal consistency was calculated with Cronbach’s α. Group differences were analyzed with Student’s *t*-test for independent samples or χ^2^ for nominal data, group differences were calculated to demonstrate effect sizes using Cohen’s *d*. Data were analyzed with IBM SPSS 24, effect sizes were calculated using an online calculator^1^.

### MRI Data Acquisition and Analysis

Data were acquired using a 3T Siemens MAGNETOM Trio system (Siemens Medical Solutions) equipped with a 12-channel head coil: structural T1-weighted image (TR: 2,530 ms, TE: 3.32 ms, flip angle: 7°, voxel size: 1 × 1 × 1 mm, field of view: 256 mm, measurements: 1), rsfMRI (TR: 2,000 ms, TE: 28 ms, flip angle: 80°, voxel size: 3 × 3 × 3 mm, field of view: 216 mm, measurements: 200), and task fMRI (TR: 2,500 ms, TE: 28 ms, flip angle: 80°, voxel size: 3 × 3 × 3 mm, field of view: 216, measurements: 364). After the rsfMRI and rumination induction tasks, B0 inhomogeneity field maps were collected (TR: 400 ms, TE: 4.5 ms/6.96 ms, flip angle: 60°, voxel size: 3 × 3 × 3 mm, field of view: 216 mm, measurements: 1).

The DICOM series were converted to NIfTI and BIDS data formats with Horos Bids Output^2^. Spatial preprocessing was performed using Statistical Parametric Mapping (SPM12^3^). Functional images were corrected for distortions related to magnetic field inhomogeneity, corrected for motion by realignment to the first acquired image, slice-timed, normalized to the MNI space, and resliced to obtain a resolution of 2 × 2 × 2 mm, and smoothed with the 6 mm FWHM Gaussian kernel. Before normalization, structural images were coregistered to the mean functional image and segmented into separate tissues using the default tissue probability maps. Functional data were also analyzed with the Artifact Detection Toolbox (ART^4^). Any EPI which deviated from the previous one by 3SD, 1.6 mm, or 0.04 rad was considered an outlier and such EPIs were regressed out in the 1st level models. Averages of 4.12%, *SD* = 2.64%, of scans for the rumination induction task and of 4.74%, *SD* = 4.13%, of scans for rsfMRI were regressed out. Participants with more than 20% outliers were excluded from the analyses. Based on these criteria no participants were excluded. There were no differences between groups in the number of outliers in the rumination induction task (*t* = 0.23, *p* = 0.82) or in the resting state (*t* = -1.76, *p* = 0.08), there were also no differences in the number of outliers between RUM and ABS conditions (*t* = 0.23, *p* = 0.82). Functional data were high pass filtered (1,000 s for rumination induction and 128 s for rsfMRI), and fixation crosses in the rumination induction task were modeled as baseline. Data were analyzed as a flexible factorial model of group × condition activation and with a two sample *t*-test of RUM > ABS and ABS > RUM contrasts. A regressor with a mock variable for gender was added to the second level models. On a group level, a voxel-wise height threshold of *p* < 0.05 corrected for multiple comparisons using the family wise error (FWE) rate was employed for whole brain analyses. Thresholded fMRI maps and raw data are available to any researcher upon request.

#### Functional Connectivity Analyses

The CONN (ver. 18^5^) toolbox was used to perform functional connectivity analyses. First level SPM files and functional data for the resting state and rumination induction were imported into the software. Data were denoised with use of the respective T1-weighted scans, normalized to MNI-space, with eight regressors for WM and seven regressors for CSF, and with movement parameters obtained with the ART toolbox. The acceptance threshold for denoised signal voxel-to-voxel correlations was on average *r* ≤ 0.1. Resting state connectivity was calculated as HRF modulated pairwise correlations with seed-to-voxel analyses with a regressor for gender. RumInd connectivity was calculated as HRF modulated pairwise regressions with seed-to-voxel analyses of the generalized psychophysiological interaction (gPPI; McLaren et al., 2012) of group (HCAS and LCAS) versus condition (RUM and ABS) interactions with a regressor for gender. To make things clearer, η^2^, the effect size for the interaction analysis, was transformed into Cohen’s *d* using an online calculator (see footnote 1). The threshold for significance was set at *p* ≤ 0.05 with false discovery rate cluster correction (FDRc). Figures depicting the connectivity analyses were made with use of MRIcroGL^6^.

#### Seed Definitions

ROIs (regions of interest) chosen for functional connectivity seeds were based on main effects of the RUM condition from the rumination induction task and analysis of meta-analytic literature on the neural correlates of emotional disorders (i.e., depression and anxiety), these being conceptually most similar to CAS activation. Spheres of *r* = 6 mm were created over the obtained peak activations or the coordinates of peak activations provided by other authors. The MarsBar toolbox^7^ was used to create ROIs. Talairach coordinates from meta-analyses were converted to MNI coordinates with the mni2tal calculator^8^. Nine ROIs were extracted from the RUM > ABS contrast from the rumination induction task: left and right precunei [-4 -58 32, -8 -52 28 and 6 -52 26], middle cingulate cortex [0 -18 36], L-paracingulate gyrus [-6 52 8], L- and R-superior frontal gyri [-2 56 38 and 6 52 28] and L- and R-frontal poles [-4 62 24 and 4 56 10]. Task-based ROI labels were based on an Harvard–Oxford anatomical atlas. Nine ROIs were extracted from meta-analyses on depressive and anxiety disorders: sub-callosal gyrus [2 16 -12], R-anterior cingulate cortex [10 30 -4] (Depression; Palmer et al., 2015), L-insula [-41 -3 -14], R-dorsal anterior cingulate cortex [-2 32 21], R-dorsolateral prefrontal cortex [30 10 50], and L-dorsolateral prefrontal cortex [-23 25 46] (Depression; Hamilton et al., 2012), L-insula [-42 14 -1] (Social anxiety disorder; Ipser et al., 2013), R-anterior cingulate [5 28 18], and R-middle frontal gyrus [41 9 40] (PTSD; Simmons and Matthews, 2012). Literature-based ROI labels were based on nomenclature used by the authors of meta-analyses. Due to the long-block nature of the rumination induction task, we limited these analyses to cortical regions chosen as ROIs.

## Results

### Behavioral Results

HCAS and LCAS groups differed strongly on all CAS measures (CAS-1, RRS-brooding, and MCQ-30 subscales) and all the subscales of SCL-27-plus used in this study. All differences were large in effect size with values of *d* > 3.5 for CAS measures and values of *d* > 1.3 for measures of psychopathology. There were more women in the HCAS group, for this reason, a mock variable for gender was added to the second levels of the fMRI and functional connectivity analyses. The groups also differed significantly with medium-to-large effect sizes on their assessments during rumination induction, both in RUM and ABS conditions as well as post-scan measurements of anxiety and negative emotions – for details see Table 2.

**Table 2:** Behavioral results of RumInd-M task and post-scan assessments.

		HCAS (*n* = 25)	LCAS (*n* = 33)	*t*-Test	*p*-Value	Cohen’s *d* (90% CI)
Modified rumination induction	RUM-sadness	4.24 (1.64)	2.09 (0.39)	6.40	<0.001	1.80 (1.29–2.32)
	RUM-anxiety	4.04 (2.07)	2.03 (0.18)	4.84	<0.001	1.37 (0.89–1.85)
	RUM-engagement	8.56 (1.76)	8.28 (2.23)	0.51	0.611	
	ABS-sadness	2.84 (1.03)	2.00 (0.00)	4.07	<0.001	1.15 (0.69–1.62)
	ABS-anxiety	3.12 (1.72)	2.03 (0.18)	3.16	0.004	0.89 (0.44–1.34)
	ABS-engagement	8.24 (1.96)	8.84 (1.59)	-1.28	0.205	
STAI-state		42.32 (11.26)	29.55 (4.64)	5.34	<0.001	1.48 (1.00–1.97)
PANAS-negative emotions		27.56 (10.51)	16.21 (1.55)	5.35	<0.001	1.51 (1.02–2.00)

*RUM, ABS, conditions in RumInd-M task; STAI, State-Trait Anxiety Inventory - State Version; PANAS, Positive and Negative Affect Schedule.*

### Neuroimaging Results

**FIGURE 3:**
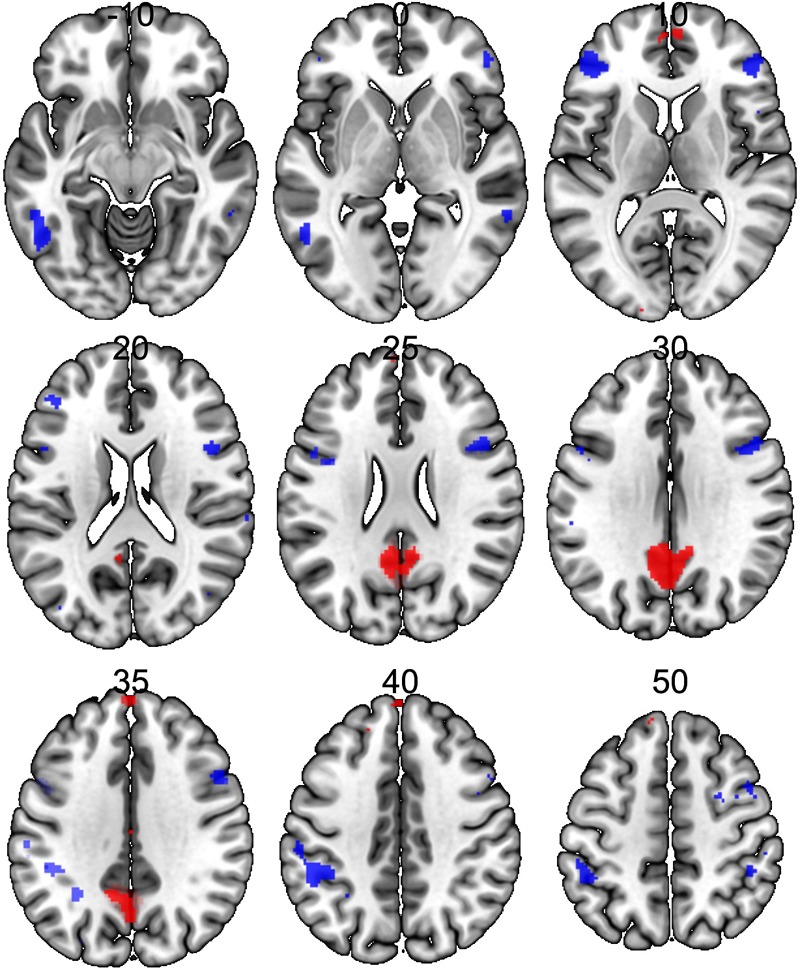
Neural activations in the whole sample (both groups together) for RUM > ABS and ABS > RUM contrasts. Red clusters depict activations in the RUM > ABS contrast, blue clusters depict activations in the ABS > RUM contrast. For details, see Table 3.

Significant neural activations in the whole sample for RUM > ABS and ABS > RUM contrasts are presented in Figure 3 and Table 3. The RUM > ABS condition yielded activations in bilateral precunei, bilateral superior frontal cortices, bilateral frontal poles, and the middle cingulate cortex. The ABS > RUM condition yielded several cortical activations: bilateral middle temporal gyri, bilateral supramarginal gyri, L-precentral gyrus, R-middle and inferior frontal gyri, and bilateral frontal poles. We did not find any differences between groups in neuronal activity in contrasts between RUM and ABS conditions in the rumination induction task, in the flexible factorial model, or in the two sample *t*-test models.

**Table 3:** Structure activations for both groups in RUM > ABS and ABS > RUM contrasts with FWE correction (p ≤ 0.05).

Structure name	Cluster size	Peak *Z*-value	MNI coordinates [*x y z*]
RUM > ABS contrast			
L-Precuneus^∗^	613	6.43	-4 -58 32
L-Precuneus^∗^	613	6.30	-8 -52 28
R-Precuneus^∗^	613	6.13	6 -52 26
L-Superior frontal gyrus	31	5.30	-2 56 38
	2	5.02	-18 40 38
R-Frontal pole	11	5.16	4 56 10
L-Paracingulate gyrus	26	5.08	-6 52 8
L-Frontal pole	7	5.03	-4 62 25
	4	4.90	-12 44 50
Middle cingulate cortex	2	4.76	0 -18 36
R-Superior frontal gyrus	1	4.83	6 52 28
ABS > RUM contrast			
L-Frontal pole	315	7.18	-46 40 12
R-Middle temporal gyrus	108	6.57	60 -56 -6
L-Middle temporal gyrus^∗^	372	6.49	-54 -56 -6
L-Inferior temporal gyrus^∗^	372	6.33	-50 -60 -14
L-Supramarginal gyrus	326	6.47	-50 -42 50
R-Frontal pole	179	6.22	48 38 4
R-Middle frontal gyrus^∗^	219	5.76	50 14 34
R-Inferior frontal gyrus^∗^	219	5.73	46 10 18
L-Middle frontal gyrus	33	5.43	-50 10 32
L-Superior parietal lobule	21	5.24	-30 -54 38
R-Middle frontal gyrus	80	5.20	40 4 58
R-Supramarginal gyrus	23	5.19	44 -40 50
L-Precentral gyrus	13	5.11	-40 2 24

*R, right hemisphere; L, left hemisphere; ^∗^one cluster containing parts of two structures.*

### gPPI Results

**FIGURE 4:**
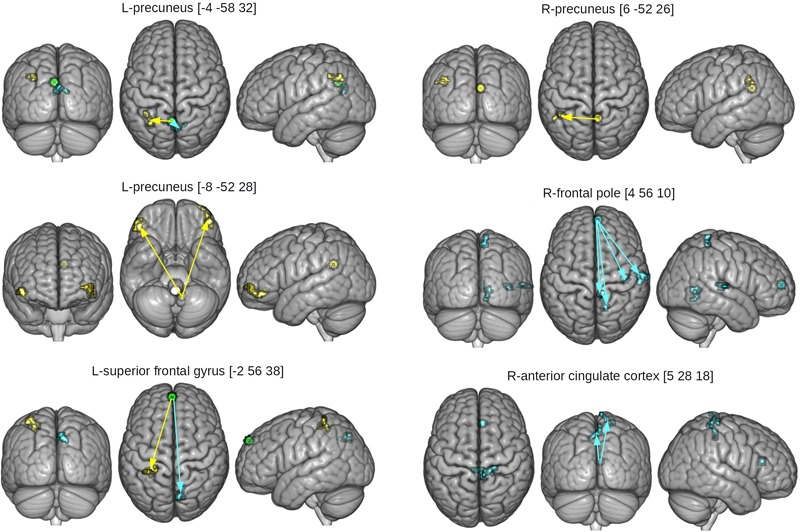
Seed and effect clusters for gPPI analyses. Yellow clusters depict increased connectivity in the HCAS group in the RUM condition and/or decreased connectivity in the ABS condition in comparison to the LCAS group, cyan clusters depict decreased connectivity in the HCAS group in the RUM condition and/or increased connectivity in the ABS condition in comparison to the LCAS group. Green clusters depict seeds with bidirectional effects. Beginnings of arrows mark the seeds and ends mark the effects. For details of seeds, see Table 4.

Table 4 and Figure 4 displays results of gPPI of group and condition interactions. The L-precuneus [-4 -58 32] showed increased connectivity with parts of the L-lateral occipital cortex and supramarginal gyrus in the HCAS group in the RUM condition in comparison to the LCAS group and decreased connectivity with bilateral parts of the precunei in the RUM condition in comparison to the LCAS group; opposite effects were observed in the ABS condition. The L-superior frontal gyrus showed decreased connectivity with parts of the L-superior parietal lobule and postcentral gyrus in the HCAS group in the ABS condition in comparison to the LCAS group and increased connectivity with the R-precuneus in this group in the ABS condition in comparison to LCAS group; opposite effects were seen in the RUM condition. Also, the L-precuneus [-8 -52 28] showed increased connectivity with bilateral frontal poles in the HCAS group in the RUM condition in comparison to the LCAS group and the opposite effect was found in the ABS condition. There was also increased connectivity in the HCAS group in the RUM condition between the R-precuneus and parts of the L-angular gyrus and supramarginal gyrus in comparison to the LCAS group; the opposite effect was observed in the ABS condition. The R-frontal pole showed decreased connectivity in the HCAS group in the RUM condition with four effect clusters in the right temporal and right parietal lobes (see Table 4 for details) in comparison to the LCAS group; opposite effects were observed in the ABS condition. A similar pattern of connectivity was observed in the R-anterior cingulate cortex and its effect clusters – bilateral precentral and R-postcentral gyri, and R-pre- and postcentral gyri. All presented interaction effects are significant with large effect sizes of Cohen’s *d* > 1.

**Table 4:** Group differences in gPPI rumination induction functional connectivity.

Seed [*x y z*]	Effect [*x y z*]	Cluster size	Peak *Z*	*p*-value for cluster FDRc	HCAS		LCAS		Cohen’s *d*
					RUM mean β	ABS mean β	RUM mean β	ABS mean β	
L-Precuneus [-4 -58 32]	L-Lateral occipital cortex, supramarginal gyrus [-30 -62 38]	114	3.91	0.014	0.11 (0.33)	-0.07 (0.35)	-0.08 (0.25)	0.15 (0.29)	1.43
	Bilateral precuneus [0 -66 22]	104	4.85	0.012	-0.11 (0.40)	0.14 (0.30)	0.08 (0.35)	-0.16 (0.31)	1.44
L-Precuneus [-8 -52 28]	L-Frontal pole [-42 58 -2]	168	4.46	0.001	0.24 (0.45)	-0.22 (0.53)	-0.29 (0.44)	-0.01 (0.43)	1.49
	R-Frontal pole [48 40 -6]	128	4.08	0.002	0.02 (0.29)	-0.21 (0.43)	-0.39 (0.41)	-0.06 (0.34)	1.55
L-Superior frontal gyrus [-2 56 38]	L-Superior parietal lobule, postcentral gyrus [-24 -38 56]	150	4.17	0.002	0.04 (0.25)	-0.21 (0.30)	-0.01 (0.28)	0.07 (0.30)	1.44
	R-Precuneus [8 -70 42]	114	4.50	0.012	-0.41 (0.44)	0.17 (0.76)	-0.25 (0.46)	-0.29 (0.54)	1.14
R-Precuneus [6 -52 26]	L-Angular gyrus, supramarginal gyrus [-42 -48 34]	86	4.46	0.041	0.05 (0.35)	-0.15 (0.18)	-0.22 (0.33)	0.03 (0.29)	1.31
R-Frontal pole [4 56 10]	R-Lingual gyrus [14 -56 0]	112	4.03	0.01	-0.20 (0.50)	-0.04 (0.32)	0.16 (0.42)	-0.14 (0.34)	1.22
	R-Planum temporale [58 -26 10]	111	4.07	0.01	-0.24 (0.38)	-0.12 (0.37)	0.09 (0.38)	0.24 (0.38)	1.22
	R-Postcentral gyrus [8 -42 62]	88	4.60	0.033	-0.26 (0.32)	-0.05 (0.37)	0.07 (0.34)	-0.17 (0.28)	1.28
	R-Heschl’s gyrus, insular cortex [38 -22 8]	84	5.17	0.041	-0.19 (0.26)	-0.08 (0.26)	0.04 (0.22)	-0.23 (0.29)	1.56
R-Anterior cingulate cortex [5 28 18]	Bilateral precentral, R-postcentral gyri [4 -32 56]	96	4.34	0.022	-0.48 (0.66)	-0.20 (0.54)	0.24 (0.39)	-0.11 (0.43)	1.53
	R-Pre–postcentral gyri [14 -32 72]	90	4.25	0.030	-0.37 (0.39)	-0.18 (0.48)	0.28 (0.57)	-0.16 (0.49)	1.44

*L, left hemisphere; R, right hemisphere; HCAS, high-CAS group; LCAS, low-CAS group; RUM, rumination condition in RumInd-M; ABS, abstract condition in RumInd-M.*

### Resting State Functional Connectivity Results

**FIGURE 5:**
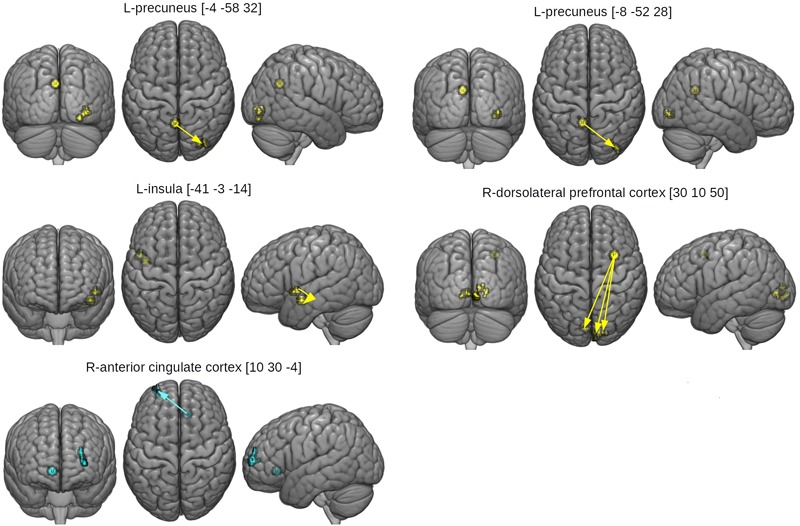
Seed and effect clusters of rsfMRI analyses. Yellow clusters depict increased connectivity in the HCAS group in comparison to the LCAS group. Cyan clusters depict decreased connectivity in the HCAS group in comparison to the LCAS group. Beginnings of arrows mark the seeds and ends mark the effects. For details, see Table 5.

The between-group differences in rsfMRI functional connectivity are presented in Table 5 and Figure 5. The HCAS group showed increased connectivity in comparison to the LCAS group between the L-insula and the L-central opercular cortex and planum temporale. Similarly, stronger connectivity in the HCAS group was found for the seed in the R-dorsolateral prefrontal cortex leading to three resulting clusters in the R-occipital pole and intracalcarine cortex, R-occipital pole and lingual gyrus, and the L-intracalcarine cortex and lingual gyrus. On the other hand, there was decreased connectivity in the HCAS group in comparison to the LCAS group between the R-anterior cingulate cortex and the L-frontal pole. All differences were large in effect with all values of *d* > 1.

**Table 5:** Group differences in resting state functional connectivity.

	Seed [*x y z*]	Effect [*x y z*]	Cluster size	Peak *Z*	*p*-Value for cluster size FDRc	HCAS mean *Z*	LCAS mean *Z*	Cohen’s *d* (CI 90%)
HCAS > LCAS	
	L-Precuneus [-4 -58 32]	R-Lateral occipital cortex, fusiform gyrus [28 -86 -12]	140	4.37	0.002	0.02 (0.09)	-0.09 (0.13)	0.98 (0.52 – 1.45)
	L-Precuneus [-8 -52 28]	R-Lateral occipital cortex [36 -84 -4]	71	3.85	0.043	0.06 (0.11)	-0.06 (0.14)	0.95 (0.49 – 1.41)
	L-Insula [-41 -3 -14]	L-Central opercular cortex [-48 4 -2]	98	4.85	0.012	0.21 (0.09)	0.11 (0.06)	1.31 (0.82 – 1.79)
	R-Dorsolateral prefrontal cortex [30 10 50]	L-Intracalcarine cortex, lingual gyrus [-8 -84 0]	100	4.31	0.010	0.05 (0.07)	-0.05 (0.11)	1.09 (0.62 – 1.55)
		R-Occipital pole, intracalcarine cortex [12 -90 6]	83	4.03	0.013	0.05 (0.07)	-0.04 (0.09)	1.12 (0.65 – 1.59)
		R-Occipital pole, lingual gyrus [6 -92 -6]	61	4.18	0.032	0.06 (0.09)	-0.05 (0.10)	1.16 (0.69 – 1.63)
LCAS > HCAS	
	R-Anterior cingulate cortex [10 30 -4]	L-Frontal pole [-32 64 6]	84	4.21	0.023	0.01 (0.07)	0.10 (0.06)	-1.38 (-1.87 – -0.90)

*L, left hemisphere; R, right hemisphere; HCAS, high-CAS group; LCAS, low-CAS group.*

## Discussion

The present study used the rumination induction fMRI task and rsfMRI method to disentangle differences in the neural functioning of people with elevated levels of CAS in comparison to people with low levels of CAS. We ensured that the groups had extreme characteristics by pre-selecting two subsamples of people with low and high results on various measures of CAS and, additionally, by excluding participants with non-extreme and inconclusive results on the day of the study. A series of self-assessment questionnaires before, during, and after the fMRI procedure was used to address different levels of CAS, psychopathology symptoms, and negative emotions.

### Group Differences in Self-Assessment

By their construction, the studied groups differed significantly on all used measures of CAS – the CAS-1 questionnaire, rumination, and metacognitive beliefs concerning the need to control thoughts as well as the perceived inability to control thoughts and the associated dangers. Nevertheless, both groups also differed in levels of psychopathology symptoms – both depressive (Papageorgiou and Wells, 2003, 2009; Fergus et al., 2012, 2013) and anxiety symptoms (Wells, 2005; Fergus et al., 2012, 2013), as well as pain symptoms. This result is in line with numerous studies on the relationships of psychopathology with somatic symptoms and complaints (Bair et al., 2003; Kroenke, 2003; Tsang et al., 2008). It is noteworthy that the groups did not differ in terms of physical illnesses and concerns reported in SCID-I (cf. Dragan and Kowalski, unpublished). The discrepancy between lack of difference in number of physical illnesses and concerns in SCID-I and large difference in self-reported levels of pain symptoms may be due to self-focused attention and threat monitoring in people with high levels of CAS, resulting in fixation of attention on bodily sensations that would otherwise go unnoticed. Such a mechanism would be consistent with an understanding of health anxiety based on the metacognitive model (Melli et al., 2018).

There were medium to large group differences in reported assessments of sadness and anxiety during rumination induction, but not in assessments of engagement. The HCAS group scored significantly higher on levels of these negative emotions not only when assessing their mood after the rumination condition but also, with smaller effect size, after reading the abstract sentences. Results from previous studies on patients with depression are mixed: in one study there were no differences in negative affect between MDD patients and controls during rumination induction despite initial differences (Berman et al., 2014), and another study (Burkhouse et al., 2017) found a significant effect of group, as remitted MDD adolescents had higher sadness ratings during both rumination and abstract conditions. Our study dealt with people with time-persistent high or low levels of CAS, so these results may indicate that CAS levels are a prominent characteristic related to experiencing negative affect during rumination induction. This could serve as an explanation of remitted MDD adolescents having higher negative affect scores at all times (Burkhouse et al., 2017) and current MDD patients (Berman et al., 2014) having such scores only initially, before rumination induction. This hypothesis needs to be verified by further studies which take these results about CAS levels into account. The large-effect group differences in levels of post-fMRI assessments of anxiety and negative emotions are also in line with this interpretation. Unfortunately, we did not collect pre-rumination-induction assessments of affect, which would enable the comparison of effects of group as well as group and time interactions.

### Effects of Negative and Abstract Thinking

The results pertaining to main effects of conditions are partially in line with previous results about rumination induction (Cooney et al., 2010). The RUM > ABS direct comparison in our study revealed neural activations in the bilateral precunei, middle cingulate cortex, L-paracingulate gyrus, bilateral superior frontal gyri, and bilateral frontal poles. Cooney et al. (2010) reported a similar pattern of activations with larger parts of the frontal cortices as well as the occipital and temporal gyri, but using a lenient statistical threshold. This indicates engagement of the DMN (Greicius et al., 2003) with the most prominent activation in both precunei (Zhang and Chiang-shan, 2012). Precuneal activity is often linked to self-referential processing (Kjaer et al., 2002; Lou et al., 2004) and depressive rumination (Johnson et al., 2009; Cooney et al., 2010; Milazzo et al., 2014; Burkhouse et al., 2017). The medial parts of the prefrontal cortex are also associated with self focused attention (Gusnard et al., 2001) and emotional responses (Lane et al., 1997). Such a pattern of activation during negative thinking induction may reflect cognitive components of negative thinking, specifically self-focused attention and self-referential processing. There were no significant brain activations in regions involved in emotional processing in the RUM > ABS comparison, i.e., in the amygdalae, parahippocampal gyri, or insulae.

Interestingly the ABS > RUM contrast (not reported by Cooney et al., 2010) revealed strong activations in the bilateral middle temporal gyri, bilateral supramarginal gyri, L-precentral gyrus, R-middle and inferior frontal gyri, L-precentral gyrus and bilateral frontal poles. Widely distributed cortical activations in parts of the frontal poles (considered functionally as the dorsolateral prefrontal cortex) and parts of the parietal lobes can be identified as parts of the CEN (Corbetta and Shulman, 2002). The activity of the CEN, in opposition to the DMN, is associated with performing cognitive tasks, attention functioning, and working memory. The CEN as well as middle temporal regions and supplementary motor areas are also part of the “task-positive network” (Fox et al., 2005), which is a net of functionally correlated regions engaged in attention and working memory. This may indicate that abstract sentences engaged participants in tasks that required their attentional resources and were cognitively demanding.

The obtained patterns of neural activity specific to negative and abstract sentences are different and emphasize cognitive differences between these two types of thinking. It is also worth noting that both the DMN and CEN are engaged in the process of mind wandering (Christoff et al., 2009). In light of our results, this may indicate that mind wandering is comprised of self-referential rumination and dwelling on abstract cognitions.

### Group Differences in Modified Rumination Induction

As rumination induction has scarcely been used to-date in fMRI studies, we based our hypotheses concerning group differences on results obtained by Cooney et al. (2010) in a group of depressed patients. We did not replicate these results, i.e., we did not uncover any significant group differences between HCAS and LCAS groups in rumination induction in the basic fMRI analysis. There may be several reasons for this. The first reason may be the very design of the rumination induction task: it is comprised of blocks of five sentences which each last 30 s and are divided by 10 s fixation crosses, which gives almost 200 s per block. This may subject the obtained data to physiological noise (Liu, 2016) or noise due to the instabilities of the magnetic field inside the scanner (Smith et al., 1999). As such, long blocks prevent the filtering of low-frequency changes in the fMRI signal. Thus, it would be recommended to use shorter blocks or event-related paradigms in future studies. The second reason may be that the sentences used in our study did not directly tap into the individual experiences of participants, but were more general, aiming to evoke rumination or worry in every person, regardless of their personal experiences. This may have resulted in weaker responses to the stimuli used. It may be expected that personalized ruminative sentences would evoke much higher responses in participants (cf. Berman et al., 2014; Burkhouse et al., 2017). Another reason may be the heterogeneity of obtained results, as high levels of CAS can manifest in different ways, with a person developing mood or anxiety disorders or comorbid disorders, producing differences on the cognitive level which could result in high variability of the fMRI signal across the whole brain. However, it is also possible that the results of Cooney et al. (2010) are not replicable. The authors used a rather liberal statistical threshold. Moreover they employed AFNI and AlphaSim software, in which a bug which elevates levels of false positive results has been identified (Eklund et al., 2016). Taking all the above into account, it is possible that in the rumination induction task used, brain activity related to repetitive negative thinking is similar in both sub-populations and potential between-group differences are not detectable with ‘static’ general linear model analysis. Thus we decided to seek possible between-group differences, delving into more dynamic temporal characteristics of brain activity, i.e., applying functional connectivity analyses.

### Generalized Psychophysiological Interactions

The results of this study provide the first evidence that high levels of CAS are related to disrupted patterns of functional neural connectivity. Moreover, the between-group differences were found not only during rumination and worry, but also in abstract thinking. We conducted a gPPI functional connectivity analysis using areas found to be active in the RUM condition as seeds as well as ROIs based on meta-analytical literature on mood and anxiety disorders. The results show disrupted functional connectivity in the HCAS group within the DMN – the precunei, the medial parts of the prefrontal cortices, and parts of the occipital cortex (Greicius et al., 2003; Zhang and Chiang-shan, 2012) – during evoked negative thoughts. This may indicate a heightened tendency toward self-referential thinking and focusing attention on the self (Raichle et al., 2001; Buckner et al., 2008). A similar pattern of functional connectivity was also found in depression and interpreted as an inability of MDD patients to down-regulate cognitive activity broadly associated with the DMN (Sheline et al., 2009).

There was also an interaction indicating a pattern of heightened connectivity in the RUM condition and/or lowered connectivity in the ABS condition in the HCAS group in comparison to the LCAS group between the L-precuneus and bilateral ventrolateral prefrontal cortices (vlPFC), which play a role in emotion processing in MDD (Keedwell et al., 2005). Furthermore the vlPFC are associated with anxiety (in primates; Agustín-Pavón et al., 2012) and, more specifically, attention bias to both threatening and neutral stimuli in anxiety and anxiety related disorders (Sylvester et al., 2012) and PTSD (Fani et al., 2012). Previous research on adolescents (Guyer et al., 2008; Monk et al., 2008) has shown that functioning of the ventrolateral prefrontal cortex may be modulated by the amygdala in social phobia and GAD. Current results suggest that the functioning of the vlPFC is modulated by disrupted functioning of the DMN, particularly the precuneus, which may “override” the regulatory role of the vlPFC in emotional processing and indicates the proneness of HCAS subjects to attention bias in self-referential processing (Wells, 2009).

We also observed a disrupted connectivity pattern in parts of the DMN during the abstract condition in the HCAS group. Interaction indicating increased connectivity was found between medial parts of the frontal cortex and R-precuneus, as well as within frontal and parietal parts of the DMN, and also within the precunei. Diminished connectivity of the anterior part of the cingulate cortex, interpreted as part of the salience network (Peters et al., 2016), with medial parts of the somatosensory cortex was found in the HCAS group in both RUM and ABS conditions, as compared to the LCAS group. A similar pattern of connectivity was also found between part of the DMN – the medial part of the prefrontal cortex (mPFC) – and the medial part of the somatosensory cortex. The rostral part of the anterior cingulate cortex (ACC), which plays a role in the symptomatology of various emotional disorders (Etkin et al., 2006), was shown to modulate the activity of the amygdala in task (Etkin et al., 2006) and resting state (Margulies et al., 2007) fMRI. Diminished connectivity between the ACC, mPFC, and somatosensory cortex in the HCAS group may indicate the mechanism of disrupted regulation of perception of bodily sensations. This result may be in line with the higher scores on the pain and vegetative symptoms subscale of the SCL-27-plus in the HCAS group. Perhaps the disrupted connectivity of the ACC, mPFC, and somatosensory cortex is related to one of the core mechanisms of CAS – heightened vigilance and monitoring for threatening stimuli, including threatening bodily sensations, which is characteristic of anxiety and anxiety-related disorders (Wells and Carter, 2001; Esteve and Camacho, 2008; Ginzburg et al., 2014).

There was also an interaction indicating a decreased connectivity pattern in the RUM condition and/or increased connectivity pattern in the ABS condition in the HCAS group in comparison to the LCAS group between part of the mPFC, part of the DMN, and R-Heschl’s gyrus, insular cortex, and R-planum temporale, which have been shown to be engaged in auditory (Storti et al., 2013) and language (Nakada et al., 2001; Buchsbaum et al., 2005) processing. These results are also consistent with diminished resting state connectivity in Heschl’s gyrus and the planum temporale in high trait-anxiety participants (Modi et al., 2015). Taking into account that Heschl’s gyrus is engaged in both task-elicited and spontaneous inner speech (Hurlburt et al., 2016), it may be hypothesized that the disrupted connectivity of the DMN, mPFC in this case, and parts of auditory and language circuitries reflects the tendency for repetitive negative thinking typical of HCAS participants (Wells, 2009).

These results may not only serve as evidence for difficulty in down-regulating DMN activity in HCAS subjects during ruminative and abstract thinking, but also suggest a more global pattern of functional connectivity during various types of thinking and diminished cognitive control (Peters et al., 2016). This conclusion is supported by higher amplitudes of changes in connectivity between conditions in the HCAS group in comparison to the control group (see beta values in Table 3). Different patterns of connectivity in the more cognitively demanding ABS condition between groups also suggests that high levels of CAS may be associated with disturbances in the performance of cognitive tasks observed in clinical groups (Austin et al., 2001; Bishop et al., 2004; Eysenck et al., 2007; Hammar and Årdal, 2009; Murrough et al., 2011), which is in line with the S-REF model and the metacognitive theory of psychological disorders (Wells and Matthews, 1994; Wells, 2009).

The described results are also in line with those showing connectivity disruptions in rsfMRI and task-based fMRI in MDD patients (Zhang et al., 2011; Sambataro et al., 2014; Palmer et al., 2015) and anxiety disorder patients (Ding et al., 2011; Lei et al., 2015). This suggests that clinical levels of psychopathology and clinical diagnoses may not be necessary to observe disrupted patterns of functional connectivity in the brain. High levels of CAS may serve as an underlying factor not only for the symptoms observed in various clinical afflictions, but also can be associated with corresponding patterns of neural functioning.

### Resting State Functional Connectivity

In the current study, we also examined functional connectivity from brain activity recorded during a 10-min-long resting state fMRI procedure. We found the HCAS group to be characterized by stronger connectivity between several brain regions as compared to the LCAS group. First, the HCAS group showed stronger functional connectivity between the posterior part of the insula, a region involved, inter alia, in emotional processing during memory retrieval (Phan et al., 2002) and part of the opercular cortex in the left hemisphere, which is associated with auditory imagery (Lima et al., 2015). This pattern of connectivity could reflect the process of repetitive negative thinking occurring in the HCAS group – with interplay between parts of brain associated with emotion processing during memory retrieval (Phan et al., 2002) and verbal imagery. Increased connectivity was also found between the R-dorsolateral prefrontal cortex, which is associated with working memory and a part of the CEN (Corbetta and Shulman, 2002), and medial parts of the occipital lobe cortex associated with word recognition and processing (Mechelli et al., 2000) and visual processing (Kozlovskiy et al., 2014). Perhaps this increased connectivity may reflect common activations of these structures on a daily basis during the frequent rumination, worry, and reflection of the participants in the HCAS group. This is consistent with the results of the questionnaires they filled-in immediately before the fMRI study. It is noteworthy that diminished, not increased, connectivity was found between frontal and occipital brain regions in patients with social anxiety disorder (Ding et al., 2011). This result was interpreted by the authors as disrupted processing of visual stimuli in social contexts. Similarly, our results may suggest that CAS is an underlying factor of the heightened salience of threatening social cues in social anxiety disorder. This calls for investigation in further studies, as the results of this and other studies are mixed.

There was also a pattern of decreased connectivity found in the HCAS group as compared to the control group. This pattern was observed between part of the ACC and part of the ventral frontal pole which, again, are parts of the salience and CENs, respectively. Disruption in this connection was found in patients with GAD and interpreted as a dysfunction of top-down control over emotion regulation (Mochcovitch et al., 2014). In general, the obtained results can be understood as altered interplay between different brain networks in people with high levels of CAS. Similar abnormalities were reported in studies on different clinical disorders such as depression (Zhang et al., 2011; Mulders et al., 2015; Peters et al., 2016) and social anxiety (Ding et al., 2011; Liu et al., 2015). This points to CAS as a probable factor underlying the clinical symptomatology and disrupted neural functional connectivity in people with different clinical afflictions, or even in people without a current diagnosis but with a high risk of developing emotional disorders.

## Conclusion

To our knowledge, this is the first study to explore the neural correlates of CAS. In this study we showed that treatment- and diagnosis-naive people with high levels of CAS differ substantially from people with low levels of this syndrome on various psychopathology and affect measures. Nearly half of the HCAS group was diagnosed with at least one current psychiatric disorder, predominantly mood and anxiety disorders as well as PTSD. We also demonstrated a large difference in self-assessment in these groups during repeated induction of negative thinking. These serve as proof-of-concept results of the metacognitive theory of emotional disorders (Wells, 2009). Contrary to our first hypothesis, we had no success in replicating rumination induction results in depressed participants (Cooney et al., 2010), for which there may be methodological and theoretical reasons. Irrespective of previous results, we demonstrated that neuronal activity during negative thinking is strongly related to neural activation of the DMN and that brain activity patterns during abstract thinking resemble the CEN. We were able to demonstrate evidence for our two hypotheses regarding differences in functional connectivity between groups. We showed, that low- and high-CAS groups differed in measures of functional connectivity during rumination and worry as well as during abstract thinking and resting state fMRI: high levels of CAS were related to disrupted patterns of connectivity within and between various brain networks – the DMN, the salience network, and the CEN. Overall, our results suggest that people with high levels of CAS tend to have disrupted neural processing in the areas of self-referential, task-oriented, and emotional processing. The obtained results are broadly analogous to results obtained in fMRI studies of different clinical groups with mood, anxiety, and PTSDs, which serves as an argument for recognizing high levels of CAS as an underlying factor of emotional disorders and their neural correlates. These results are consistent with the theoretical underpinnings of the metacognitive theory of psychopathology, suggesting a common mechanism of emotional disorders originating in CAS and laying the foundations for further exploration of neural correlates of CAS. Future studies should use different, better-established fMRI paradigms and more differentiated groups, such as people with high levels of CAS with and without clinical diagnoses.

## Author Contributions

JK, MW, AM, and MD wrote the manuscript. JK and MD conducted the research. JK, MW, and AM analyzed the MRI data. MW and AM supervised the MRI part of the study. MD supervised the research and analyses.

## Conflict of Interest Statement

The authors declare that the research was conducted in the absence of any commercial or financial relationships that could be construed as a potential conflict of interest.

## Footnotes

1 https://www.psychometrica.de/effect_size.html

2 https://github.com/mslw/horos-bids-output

3 http://www.fil.ion.ucl.ac.uk/spm/

4 https://www.nitrc.org/projects/artifact_detect

5 https://www.nitrc.org/projects/conn

6 https://www.mccauslandcenter.sc.edu/mricrogl/

7 http://marsbar.sourceforge.net/

8 http://sprout022.sprout.yale.edu/mni2tal/mni2tal.html
