# Supplementary material for: Neural Correlates of Cognitive-Attentional Syndrome: An fMRI Study on Repetitive Negative Thinking Induction and Resting State Functional Connectivity
Source: Front Psychol. 2019 Mar 26;10:648. doi: 10.3389/fpsyg.2019.00648 (PMC6443848; doi:10.3389/fpsyg.2019.00648)
Supplement: Supplementary file 1 [file Data_Sheet_1.doc]

English translation of stimuli for RumInd-M task

Version 1

Rumination condition:

Think about why you react the way you do.

Think intensely about a matter that worries you.

Think about the kind of person you think you should be.

Think about your ability to have an intelligent public conversation.

Think about think about the expectations people have for you.

Think about the way you feel and if you can understand your feelings.

Think about what worries you about your health.

Think about how similar/different you are relative to other people.

Think about why things turn out the way they do.

Think about the problem of maintaining your current status.

Abstract condition:

Think about the change of seasons.

Think about why you like the books you do.

Think about how a plant grows.

Think about what makes a joke funny.

Think about what contributes to team spirit.

Think about the taste of chocolate.

Think about the feeling of a pair of shoes that fit well.

Think about how someone develops common sense.

Think about the quality of Krystyna Czubówna’s voice.

Think about what it feels like to throw a ball.

Version 2

Rumination condition:

Think about what people notice about your personality.

Think about the opportunities you didn’t take in your life.

Think about in what other people are better than you.

Think about your character and who you strive to be.

Think about what worries you about your future.

Think about how do you feel at the moment.

Think about how other people can judge you.

Think about understanding who you are.

Think about what constitutes your value as a person.

Think about whether you have accomplished a lot so far.

Abstract condition:

Think about the quality of Piotr Fronczewski’s voice.

Think about the sounds that campfire makes.

Think about why you like the music you do.

Think about the feeling of combed hair.

Think about what contributes to teamwork.

Think about what contributes to sense of rhythm.

Think about the feeling of being submerged in warm water.

Think about the distribution of roads near your living place.

Think about how clouds are formed.

Think about what makes a colour feel likeable.
